# Dataset on the diversity of plant-parasitic nematodes in cultivated olive trees in southern Spain

**DOI:** 10.1016/j.dib.2019.104658

**Published:** 2019-10-15

**Authors:** Antonio Archidona-Yuste, Thorsten Wiegand, Pablo Castillo, Juan A. Navas-Cortés

**Affiliations:** a Instituto de Agricultura Sostenible (IAS), Consejo Superior de Investigaciones Científicas (CSIC), Avenida Menéndez Pidal s/n, 14004, Córdoba, Spain; b Department of Ecological Modelling, Helmholtz Centre for Environmental Research - UFZ, Permoserstrasse 15, 04318, Leipzig, Germany; c German Centre for Integrative Biodiversity Research (iDiv) Halle-Jena-Leipzig, Deutscher Platz 5e, 04103, Leipzig, Germany

**Keywords:** Nematode diversity, Plant-parasitic nematodes, Cultivated olive trees

## Abstract

Datasets presented here were employed in the main work “Spatial structure and soil properties shape local community structure of plant-parasitic nematodes in cultivated olive trees in southern Spain” Archidona-Yuste et al., 2020. In this research, we aimed to unravel the diversity of plant-parasitic nematodes (PPN) associated with cultivated olive (*Olea europaea* subsp. *europaea* var. *europaea*) in southern Spain, Andalusia. The olive growing area of Andalusia is of high agriculture and socio-economic importance with an extensive distribution of this crop. To this end, we conducted a systematic survey comprising 376 commercial olive orchards covering the diversity of cropping systems applied. Data showed 128 species of PPN belonging to 38 genera and to 13 families. In addition, an extensive data set regarding to potential factors in structuring the community patterns of PPN found in the 376 commercial olive orchards sampled is provided. Three variables data set were compiled including above-ground environment, soil and agronomic management. Overall, 48 explanatory variables were selected as determinist processes on shaping the diversity of PPN. Finally, data also showed the values regarding to the partition of beta diversity into contributions of single sites to overall beta diversity (LCBD) and intro contributions of individual species to overall beta diversity (SCBD). Data may serve as benchmarks for other groups working in the field of PPN diversity associated with crops and of belowground communities and ecosystems.

**Table:** Specifications Table

Subject area	Ecology
More specific subject area	Plant-parasitic nematode ecology. A case of study: cultivated olive trees in southern Spain
Type of data	Tables and figures
How data was acquired	Nematode identification was acquired by using integrative taxonomy (using a Zeiss III compound microscope with Nomarski differential interference contrast at up to × 1000 magnification and molecular methods standardized). Variable data sets were compiled from GIS, directly provided by landowner and/or data collection
Data format	Raw and analyzed
Experimental factors	Soil samples were collected with a hoe from four to five trees randomly selected in each commercial olive orchard for both taxa identification and explanatory variables data collection.
Experimental features	Evaluate diversity, prevalence and abundance of plant-parasitic nematodes infesting soils from cultivated olive trees in southern Spain.
Data source location	Andalusia, southern Spain. Coordinates of sampling points are provided.
Data accessibility	Data is provided in this article, and raw data as supplementary material.
Related research article	Archidona-Yuste A., Wiegand T., Castillo P., and Navas-Cortés J. A. 2020. Spatial structure and soil properties shape local community structure of plant-parasitic nematodes in cultivated olive trees in southern Spain. Submitted to: Agriculture, Ecosystems and Environment, 287 (1), https://doi.org/10.1016/j.agee.2019.106688

**Table:** 

**Value of the Data** • Data may serve as benchmarks for other groups working in the field of PPN diversity infesting soils from agricultural ecosystems, and for belowground communities and ecosystems. • Data are based on the systematic survey with the largest sampling effort done on cultivated olive to date. • Data show a species list of PPN attacking to cultivated olive. Data increase the number of PPN associated with olive trees, being estimated in about 250 species documented worldwide

## 1 Data

**Fig. 1:**
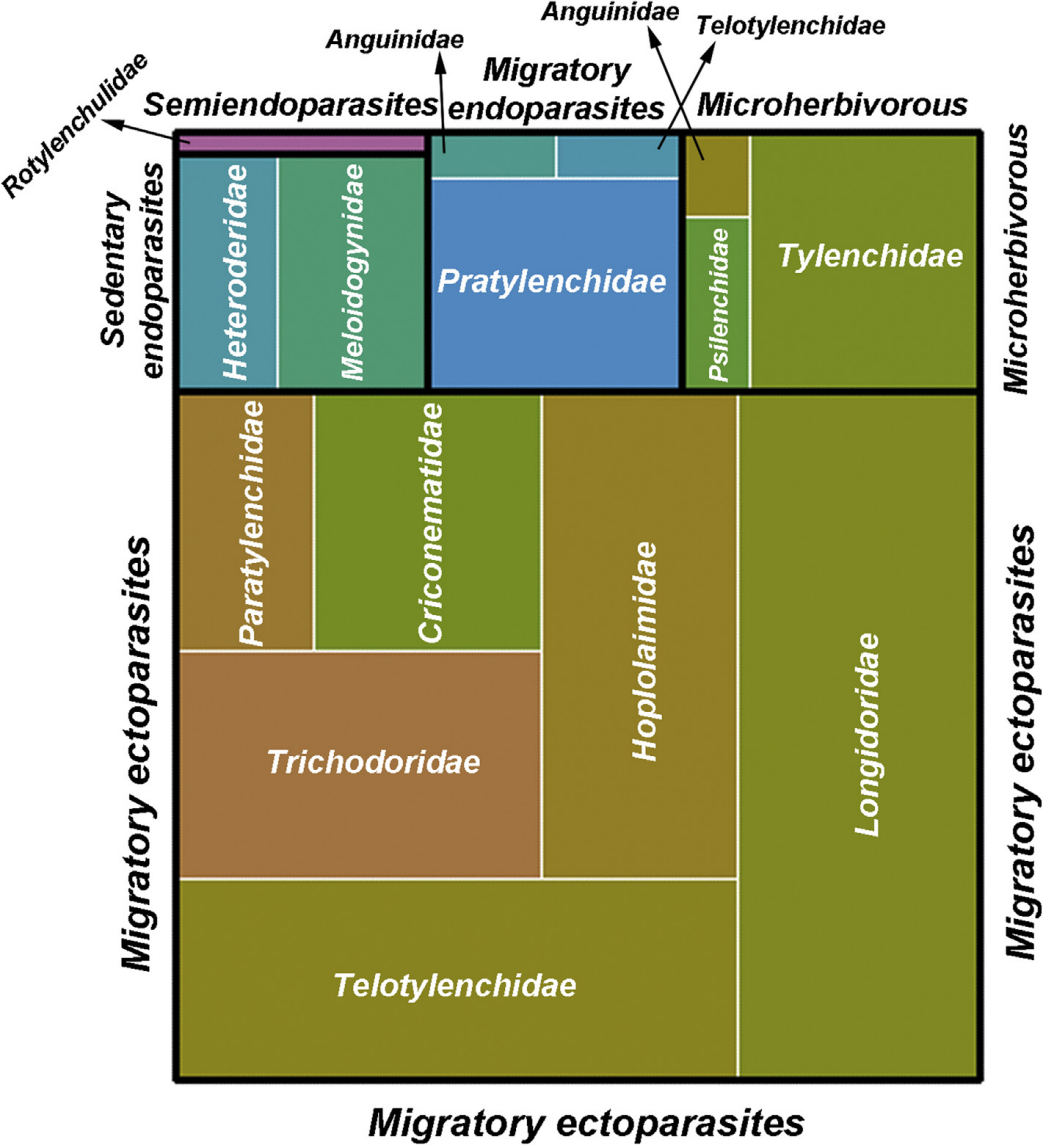
Diversity of PPN associated with cultivated olive trees in southern Spain. Tree map chart representing the diversity among feeding habits (black squares) and families (white chart) of PPN. The size of squares represents the number of taxa included in the feeding habit and/or family of PPN.

**Fig. 2:**
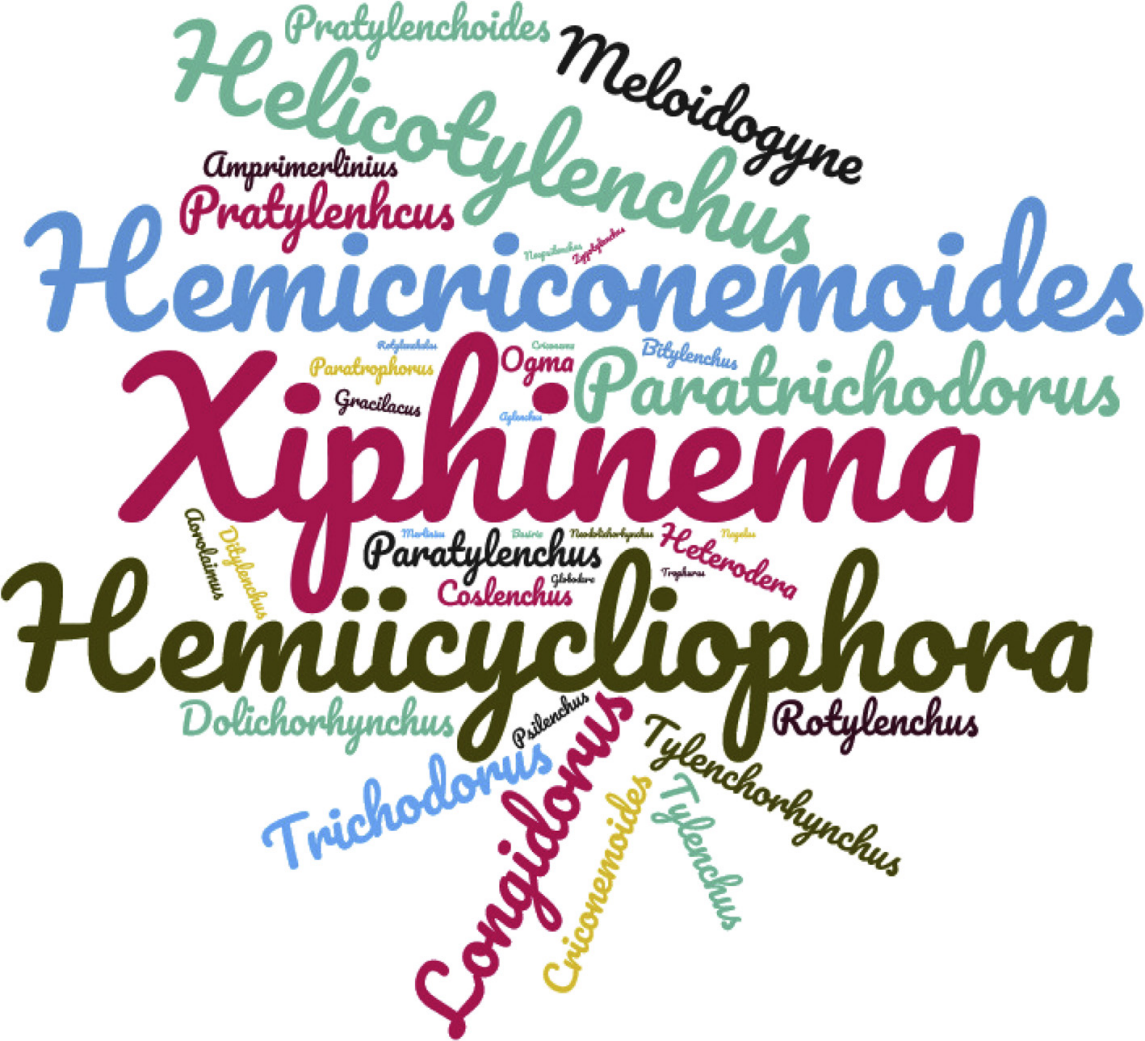
World cloud considering the genera of PPN associated with cultivated olive trees in southern Spain. The size of word indicates the number of species associated with each PPN genus.

The data presented in this article include the information of the 376 commercial olive orchards sampled, as well as the total abundance of nematodes and species richness for each commercial orchard in Table 1, information about the diversity of PPN found from the systematic survey performed in Table 2, Fig. 1, Fig. 2 In addition, Fig. 1 showed the distribution of species diversity of PPN detected by classes including feeding habit and family. Finally, values of Local Contributions to Beta diversity (LCBD) and Species Contributions to Beta Diversity (SCBD) indexes are provided in Table 1, Table 2, respectively. Table 2 showed the 27 commercial olive orchards with significant values as described by Archidona-Yuste et al. [1].

**Table 1:** Olive orchards from cultivated olive in Andalusia (southern Spain) for detecting plant-parasitic nematodes. Olive growing areas in Andalusia have been classified into 70 biologically homogeneous zones based on environmental similarities [11]. Based on these zones, 376 commercial olive orchards were selected for this study. This was done in a way that the number of sampled olive orchards per biological zone was proportional to the total olive area in each zone.

Olive orchard code	Locality, province	Latitude	Longitude	Altitude^a^	RICHNESS	Abundance	LCBD^b^
O1	Hinojos, Huelva	37°15′31.6″N	6°22′22.4″W	55	12	541	0.0039501*
O2	Hinojos, Huelva	37°20′57.9″N	6°23′01.5″W	121	6	73	0.0033041
O3	Escacena del Campo, Huelva	37°24′06.5″N	6°22′28.0″W	130	9	177	0.0028959
O4	Villalba del Alcor, Huelva	37°20′46.0″N	6°26′29.3″W	125	8	263	0.0033954
O5	Almonte, Huelva	37°14′19.3″N	6°28′58.7″W	65	8	255	0.0035093
**O6**	**Villalba del Alcor, Huelva**	**37°24′03.6″N**	**6°29′46.1″W**	**96**	**8**	**717**	0.0043700*
O7	Niebla, Huelva	37°24′04.3″N	6°42′45.1″W	101	7	98	0.0026263
O8	Niebla, Huelva	37°21′57.9″N	6°43′45.0″W	64	4	37	0.0032298
O9	Jerez de la Frontera, Cádiz	36°48′12.6″N	5°59′40.7″W	78	5	107	0.0027860
O10	Jerez de la Frontera, Cádiz	36°46′08.2″N	5°59′45.5″W	69	5	127	0.0030383
O11	Jerez de la Frontera, Cádiz	36°39′32.9″N	6°02′06.2″W	103	4	50	0.0036332
O12	Jerez de la Frontera, Cádiz	36°40′23.1″N	6°07′20.3″W	58	8	1468	0.0023036
**O13**	**Villaviciosa de Córdoba, Córdoba**	**38°2′52.65″N**	**5°0′43.18″W**	**494**	**10**	**630**	0.0039054*
O14	Belmez, Córdoba	38°14′17.5″N	5°07′16.7″W	509	5	32	0.0028062
O15	Belmez, Córdoba	38°14′33.8″N	5°08′36.9″W	513	9	139	0.0029163
O16	Fuente Obejuna, Córdoba	38°17′29.3″N	5°19′16.9″W	590	8	162	0.0037636
O17	Fuente Obejuna, Córdoba	38°15′56.1″N	5°24′55.2″W	562	6	176	0.0034448
O18	La Granjuela, Córdoba	38°22′33.9″N	5°20′46.9″W	630	10	210	0.0035947
O19	La Granjuela, Córdoba	38°22′45.5″N	5°19′27.2″W	550	3	219	0.0036349
O20	Hinojosa del Duque, Córdoba	38°24′19.0″N	5°18′10.8″W	570	10	268	0.0025421
O21	Hinojosa del Duque, Córdoba	38°24′40.7″N	5°13′06.9″W	527	7	138	0.0023221
O22	El Viso, Córdoba	38°29′56.0″N	4°58′38.4″W	623	7	167	0.0015419
O23	Alcaracejos, Córdoba	38°22′55.5″N	4°57′32.9″W	727	9	287	0.0034716
O24	Alcaracejos, Córdoba	38°15′49.0″N	4°58′47.86″W	570	8	229	0.0028375
O25	Villaharta, Córdoba	38°8′23.97″N	4°52′50.46″W	303	7	75	0.0017119
O26	Cañete de las Torres, Córdoba	37°52′31.3″N	4°20′25.1″W	341	4	136	0.0014172
O27	Porcuna, Jaén	37°52′54.3″N	4°11′29.5″W	374	6	291	0.0013965
O28	Porcuna, Jaén	37°53′44.0″N	4°08′14.3″W	229	4	187	0.0029693
O29	Andújar, Jaén	38°00′07.1″N	4°03′21.5″W	478	5	251	0.0023402
O30	Andújar, Jaén	38°07′17.0″N	3°57′41.4″W	419	6	39	0.0014365
O31	Andújar, Jaén	38°05′46.2″N	3°58′18.6″W	259	3	7	0.0024413
O32	Andújar, Jaén	38°03′49.7″N	4°00′16.7″W	191	5	149	0.0025519
O33	Marmolejo, Jaén	38°03′11.5″N	4°11′25.6″W	283	3	157	0.0027722
O34	Marmolejo, Jaén	38°03′42.0″N	4°13′24.2″W	348	4	142	0.0028993
O35	Montoro, Córdoba	38°05′59.5″N	4°16′28.3″W	452	3	28	0.0032918
O36	Montoro, Córdoba	38°07′18.9″N	4°16′44.8″W	422	7	356	0.0032150
**O37**	**Iznajar, Córdoba**	**37°15′39.1″N**	**4°19′20.0″W**	**448**	**8**	**140**	0.0040370*
O38	Prado del Rey, Cádiz	36°47′17.4″N	5°33′45.00″W	89	8	133	0.0029531
**O39**	**Rociana del Condado, Huelva**	**37°16′45.8″N**	**6°37′20.4″W**	**539**	**4**	**213**	0.0040512*
O40	Antequera, Málaga	37°08′36.0″N	4°31′28.8″W	212	6	199	0.0019654
O41	Antequera, Málaga	37°10′27.7″N	4°34′58.1″W	212	5	1380	0.0013928
O42	Mollina, Málaga	37°09′54.4″N	4°41′12.9″W	393	5	1589	0.0021571
O43	Antequera, Málaga	37°02′38.5″N	4°40′05.9″W	389	11	474	0.0021974
O44	Antequera, Málaga	37°01′48.9″N	4°45′30.2″W	470	8	846	0.0018290
O45	Campillos, Málaga	37°00′09.7″N	4°50′42.2″W	383	8	1444	0.0015922
O46	Ardales, Málaga	36°52′53.1″N	4°50′33.9″W	160	7	699	0.0014612
O47	Alora, Málaga	36°48′03.5″N	4°45′13.4″W	200	7	168	0.0017819
O48	Casarabonela, Málaga	36°46′13.8″N	4°46′54.7″W	368	6	461	0.0029229
O49	Casarabonela, Málaga	36°46′01.5″N	4°49′52.9″W	224	10	237	0.0023574
O50	Tolox, Málaga	36°41′58.5″N	4°52′46.6″W	317	8	448	0.0019210
O51	Monda, Málaga	36°38′13.0″N	4°48′24.2″W	409	8	515	0.0030593
O52	Monda, Málaga	36°37′03.0″N	4°51′00.8″W	51	5	61	0.0013762
O53	Marbella, Málaga	36°30′47.0″N	4°50′02.1″W	505	10	475	0.0028430
O54	Espiel, Córdoba	38°10′58.0″N	5°2′15.31″W	566	9	550	0.0023952
O55	Espiel, Córdoba	38°10′25.7″N	5°05′01.0″W	712	11	499	0.0026249
O56	Espiel, Córdoba	38°06′28.5″N	5°04′19.5″W	729	7	401	0.0028581
O57	Espiel, Córdoba	38°03′02.7″N	4°56′49.3″W	580	7	115	0.0024678
O58	Espiel, Córdoba	37°54′17.4″N	6°16′32.1″W	531	9	1163	0.0024123
O59	Santa Olalla del Cala, Huelva	37°53′59.1″N	6°15′22.5″W	200	10	951	0.0035445
O60	Córdoba, Córdoba	37°55′22.3″N	4°45′56.5″W	430	8	173	0.0025667
O61	Montilla, Córdoba	37°32′57.5″N	4°34′11.6″W	249	3	196	0.0015881
O62	Cabra, Córdoba	37°30′55.2″N	4°29′20.7″W	177	8	479	0.0015906
O63	Cabra, Córdoba	37°29′08.9″N	4°25′55.1″W	615	8	112	0.0014784
O64	Zuheros, Córdoba	37°32′41.1″N	4°20′47.1″W	834	3	443	0.0034653
O65	Zuheros, Córdoba	37°32′06.3″N	4°18′38.3″W	647	9	705	0.0033046
O66	Zuheros, Córdoba	37°32′49.1″N	4°18′42.6″W	551	5	264	0.0014094
O67	Luque, Córdoba	37°31′03.9″N	4°12′53.6″W	421	7	518	0.0019085
O68	Luque, Córdoba	37°34′49.4″N	4°12′38.7″W	278	6	145	0.0032546
O69	Baena, Córdoba	37°40′31.8″N	4°23′39.9″W	309	5	1399	0.0035135
O70	Baena, Córdoba	37°48′50.8″N	4°18′27.5″W	295	6	129	0.0032498
O71	Alozaina, Málaga	36°43′28.9″N	4°51′06.1″W	77	6	220	0.0012781
O72	Marbella, Málaga	36°29′53.9″N	5°00′40.1″W	180	5	384	0.0018494
O73	Paterna del Campo, Huelva	37°28′50.8″N	6°29′19.5″W	810	9	454	0.0023530
O74	Alhendín, Granada	37°04′22.3″N	3°40′40.3″W	439	5	6907	0.0022341
O75	Lecrín, Granada	36°54′37.8″N	3°31′58.3″W	665	6	358	0.0028362
O76	Lanjarón, Granada	36°54′51.3″N	3°30′01.0″W	639	7	1095	0.0026383
O77	Lanjarón, Granada	36°54′27.8″N	3°27′38.2″W	611	5	306	0.0037434
O78	Lobres, Granada	36°54′41.4″N	3°12′50.5″W	717	4	78	0.0017682
O79	Torvizcón, Granada	36°53′04.0″N	3°18′14.0″W	757	5	859	0.0021787
O80	Alcolea, Almería	36°57′48.9″N	2°57′37.9″W	963	7	1418	0.0024528
O81	Laujar de Andarax, Almería	36°58′21.4″N	2°55′22.1″W	359	4	6990	0.0023231
O82	Instinción, Almería	36°59′43.1″N	2°39′14.5″W	719	5	502	0.0029016
O83	Alicún, Almería	36°56′40.4″N	2°36′03.1″W	877	3	68	0.0014942
O84	Enix, Almería	36°52′57.8″N	2°37′28.8″W	500	6	1410	0.0021515
**O85**	**Tabernas, Almería**	**37°04′18.1″N**	**2°18′18.4″W**	**524**	**4**	**226**	0.0044046*
O86	Lucainena de las Torres, Almería	37°04′50.1″N	2°11′54.3″W	500	6	4040	0.0024570
O87	Tabernas, Almería	37°05′04.7″N	2°14′33.4″W	421	6	508	0.0031701
O88	Sorbas, Almería	37°06′22.1″N	2°08′35.7″W	593	5	371	0.0023451
O89	Uleila del Campo, Almería	37°11′08.6″N	2°11′49.6″W	252	4	174	0.0021843
O90	Cádiar, Granada	36°56′54.3″N	3°07′14.5″W	329	9	279	0.0014578
O91	Úbeda, Jaén	37°55′00.4″N	3°21′05.1″W	688	11	367	0.0025861
O92	Jódar, Jaén	37°49′10.6″N	3°19′38.8″W	698	4	1352	0.0034362
O93	Bedmar y Garcíez, Jaén	37°47′57.0″N	3°22′29.8″W	706	5	423	0.0023785
O94	Belmez de la Moraleda, Jaén	37°44′43.6″N	3°21′36.4″W	814	3	226	0.0028352
O95	Huelma, Jaén	37°43′50.0″N	3°18′00.0″W	1069	3	26	0.0012897
O96	Huelma, Jaén	37°40′09.9″N	3°18′27.9″W	1019	2	37	0.0020516
O97	Cabra del Santo Cristo, Jaén	37°39′31.0″N	3°14′14.7″W	591	6	2270	0.0020565
O98	Dehesas de Guadix, Granada	37°34′16.8″N	3°04′17.0″W	1061	3	80	0.0021005
O99	Pedro Martínez, Granada	37°32′07.4″N	3°10′52.6″W	1044	6	1147	0.0021356
O100	Pedro Martínez, Granada	37°30′38.0″N	3°12′53.3″W	1041	6	1512	0.0017842
O101	Morelábor, Granada	37°28′06.3″N	3°17′20.5″W	1092	6	489	0.0016553
O102	Morelábor, Granada	37°25′51.5″N	3°20′13.5″W	1061	5	1007	0.0014642
O103	Piñar, Granada	37°23′17.0″N	3°24′47.1″W	182	5	235	0.0013437
O104	Écija, Sevilla	37°31′04.7″N	5°09′50.0″W	164	5	577	0.0022463
O105	Fuentes de Andalucía, Sevilla	37°30′21.5″N	5°24′29.3″W	169	4	47	0.0032955
O106	Carmona, Sevilla	37°29′41.6″N	5°28′33.0″W	123	5	84	0.0038680
O107	Carmona, Sevilla	37°28′37.4″N	5°42′26.7″W	139	6	1731	0.0024029
O108	Olivares, Sevilla	37°25′51.2″N	6°08′11.9″W	30	4	1666	0.0028651
O109	Sanlúcar la Mayor, Sevilla	37°23′49.9″N	6°13′20.3″W	30	4	3414	0.0021621
O110	Sanlúcar la Mayor, Sevilla	37°23′49.0″N	6°13′19.3″W	96	8	765	0.0022083
O111	Sanlúcar la Mayor, Sevilla	37°30′50.2″N	6°12′33.5″W	56	2	50	0.0036413
O112	Guillena, Sevilla	37°33′56.2″N	6°02′33.9″W	53	3	24	0.0031674
O113	Villaverde del Río, Sevilla	37°36′22.1″N	5°54′25.5″W	395	3	937	0.0023797
O114	Villaverde del Río y Minas, Sevilla	37°39′51.4″N	5°44′56.7″W	46	7	7444	0.0038494
**O115**	**Lora del Río, Sevilla**	**37°37′41.0″N**	**5°40′29.4″W**	**129**	**3**	**365**	0.0042194*
**O116**	**Alcolea del Río, Sevilla**	**37°37′50.5″N**	**5°35′01.4″W**	**65**	**5**	**72**	0.0042684*
**O117**	**La Campana, Sevilla**	**37°35′21.4″N**	**5°27′36.4″W**	**136**	**3**	**45**	0.0042099*
**O118**	**La Campana, Sevilla**	**37°36′24.0″N**	**5°23′18.9″W**	**46**	**5**	**323**	0.0039655*
**O119**	**Utrera, Sevilla**	**37°09′12.2″N**	**5°46′39.9″W**	**24**	**8**	**288**	0.0040418*
O120	Utrera, Sevilla	37°04′36.2″N	5°48′37.8″W	67	7	394	0.0017599
O121	Utrera, Sevilla	36°59′22.6″N	5°49′39.9″W	133	5	1773	0.0018613
O122	Utrera, Sevilla	36°56′37.7″N	5°48′25.2″W	45	7	2404	0.0018732
O123	Arcos de la Frontera, Cádiz	36°44′32.3″N	5°51′32.5″W	71	4	996	0.0023408
O124	Arcos de la Frontera, Cádiz	36°40′35.0″N	5°51′12.8″W	175	5	12	0.0016646
O125	Arcos de la Frontera, Cádiz	36°35′50.2″N	5°47′08.2″W	10	9	956	0.0027686
**O126**	**San José del Valle, Cádiz**	**36°40′44.9″N**	**5°27′11.0″W**	**140**	**8**	**611**	0.0040427*
O127	Ubrique, Cádiz	36°52′43.0″N	5°34′31.5″W	167	5	718	0.0024564
O128	Constantina, Sevilla	37°52′56.4″N	5°38′06.7″W	138	5	60	0.0024440
O129	Santa Cruz, Córdoba	37°57′37.2″N	4°27′56.8″W	135	5	709	0.0027089
O130	Ecija, Sevilla	37°31′45.9″N	4°57′52.6″W	140	6	845	0.0023221
O131	Maro, Málaga	36°44′50.5″N	3°47′12.1″W	108	7	436	0.0025161
O132	Velez Málaga, Málaga	36°46′15.8″N	4°05′22.9″W	73	7	143	0.0023267
O133	Canillas de Aceituno, Málaga	36°50′41.5″N	4°07′29.7″W	172	4	529	0.0020247
O134	Alcaucín, Málaga	36°56′27.8″N	4°07′36.4″W	678	5	247	0.0021700
O135	Alfarnate, Málaga	37°00′35.3″N	4°13′55.9″W	963	5	116	0.0026151
O136	Loja, Granada	37°03′14.7″N	4°14′47.9″W	267	6	235	0.0026386
O137	Loja, Granada	37°08′40.0″N	4°12′51.9″W	177	4	939	0.0022364
**O138**	**Castillo de las Guardas, Sevilla**	**37°42′39.2″N**	**6°18′45.5″W**	**301**	**8**	**98**	0.0039617*
O139	Higuera de la Sierra, Huelva	37°50′36.0″N	6°27′32.2″W	636	7	1085	0.0038587
O140	Aracena, Huelva	37°52′15.0″N	6°30′30.1″W	585	5	98	0.0033139
O141	Aracena, Huelva	37°53′10.2″N	6°33′10.5″W	694	6	59	0.0033554
O142	Fuenteheridos, Huelva	37°55′17.7″N	6°40′03.5″W	657	6	263	0.0030968
O143	Cortegana, Huelva	37°55′40.2″N	6°49′27.1″W	664	5	59	0.0032660
O144	Aroche, Huelva	37°55′45.5″N	6°57′11.8″W	526	5	150	0.0036269
O145	Rosal de la Frontera, Huelva	37°58′32.5″N	7°11′18.5″W	208	6	230	0.0020732
O146	Rosal de la Frontera, Huelva	37°57′52.7″N	7°14′03.5″W	220	8	159	0.0026179
O147	Mengíbar, Jaén	38°1′21.70″N	3°46′38.68″W	279	5	382	0.0022702
O148	Luque, Córdoba	37°34′30.9″N	4°10′37.8″W	443	6	164	0.0030759
O149	Jerez de la Frontera, Cádiz	36°48′10.3″N	6°10′25.5″W	53	10	422	0.0035536
O150	La Rambla, Córdoba	37°37′24.3″N	4°42′17.3″W	366	8	117	0.0035630
O151	La Rambla, Córdoba	37°37′42.6″N	4°42′50.2″W	377	7	202	0.0021412
O152	Marchena, Sevilla	37°20′19.7″N	5°18′27.6″W	141	7	281	0.0014025
O153	Marchena, Sevilla	37°18′28.2″N	5°23′38.1″W	151	14	196	0.0028052
O154	Riogordo,Málaga	36°55′20.3″N	4°17′10.0″W	437	9	224	0.0023030
**O155**	**Tabernas, Almería**	**37°06′19.5″N**	**2°17′09.9″W**	**549**	**6**	**980**	0.0041281*
O156	Albaricoques-Níjar, Almería	36°51′26.6″N	2°06′08.1″W	165	5	49	0.0038506
O157	Coto Ríos, Jaén	38°01′49.4″N	2°52′05.2″W	687	10	309	0.0021103
O158	Santa M^a^ de Trassierra, Córdoba	37°54′46.0″N	4°50′53.8″W	437	8	103	0.0037143
O159	Castillo de Locubín, Jaén	37°34′28.6″N	3°58′59.9″W	718	6	169	0.0017382
O160	Morón de la Frontera, Sevilla	37°05′16.0″N	5°30′38.4″W	193	6	913	0.0027212
O161	Pozo Alcón, Jaén	37°43′47.6″N	2°55′18.0″W	948	3	1173	0.0034160
O162	Santa M^a^ de Trassierra, Córdoba	37°55′12.4″N	4°52′33.5″W	558	9	434	0.0022608
O163	Córdoba, Córdoba	37°55′18.8″N	4°45′56.4″W	197	11	390	0.0024856
O164	Bedmar y Garcíez, Jaén	37°47′52.1″N	3°22′22.6″W	720	5	3089	0.0038138
O165	Nueva Carteya, Córdoba	37°35′49.6″N	4°29′42.6″W	384	6	177	0.0034460
**O166**	**Cabra, Córdoba**	**37°30′35.9″N**	**4°31′30.9″W**	**414**	**6**	**2379**	0.0042721*
O167	Espejo, Córdoba	37°40′19.9″N	4°32′39.8″W	331	6	139	0.0021533
O168	Lucena, Córdoba	37°26′18.7″N	4°32′35.5″W	421	8	164	0.0028766
O169	Montemayor, Córdoba	37°40′40.4″N	4°41′03.5″W	251	6	48	0.0026783
O170	La Rambla, Córdoba	37°39′24.4″N	4°46′29.1″W	245	5	245	0.0026092
O171	Montalbán de Córdoba, Córdoba	37°34′01.8″N	4°46′19.9″W	169	4	231	0.0028817
O172	Santaella, Córdoba	37°29′58.0″N	4°46′54.0″W	240	5	4342	0.0030187
**O173**	**Puente Genil, Córdoba**	**37°23′02.0″N**	**4°45′54.5″W**	**212**	**5**	**7599**	0.0039211*
**O174**	**Gibraleón, Huelva**	**37°20′40.4″N**	**7°02′05.1″W**	**59**	**10**	**10071**	0.0040633*
**O175**	**Gibraleón, Huelva**	**37°24′12.4″N**	**7°00′41.1″W**	**73**	**5**	**38**	0.0042649*
O176	Trigueros, Huelva	37°23′38.1″N	6°49′57.1″W	91	9	470	0.0023190
O177	Trigueros, Huelva	37°21′57.6″N	6°49′04.9″W	61	8	79	0.0022065
O178	Niebla, Huelva	37°21′54.1″N	6°39′06.4″W	59	13	176	0.0034490
O179	La Palma del Condado, Huelva	37°23′16.9″N	6°34′49.4″W	99	6	37	0.0027454
**O180**	**Hinojos, Huelva**	**37°18′16.5″N**	**6°22′24.4″W**	**83**	**10**	**10605**	0.0041028*
O181	Hinojos, Huelva	37°18′25.8″N	6°20′37.8″W	73	7	949	0.0034245
O182	Ecija, Sevilla	37°35′42.6″N	4°58′26.8″W	234	5	65	0.0022291
O183	Marchena, Sevilla	37°20′26.6″N	5°18′23.5″W	140	7	281	0.0014025
O184	La Puebla de Cazalla, Sevilla	37°14′08.8″N	5°15′30.8″W	183	5	105	0.0033053
O185	Osuna, Sevilla	37°13′57.0″N	5°10′49.2″W	217	6	253	0.0026018
O186	Osuna, Sevilla	37°12′35.6″N	5°07′55.0″W	266	4	202	0.0029392
O187	Osuna, Sevilla	37°09′10.0″N	5°06′37.5″W	466	7	1976	0.0017018
O188	El Saucejo, Sevilla	37°05′44.2″N	5°05′30.0″W	501	5	2210	0.0017913
O189	El Saucejo, Sevilla	37°05′01.7″N	5°06′53.4″W	464	5	668	0.0037886
O190	Córdoba, Córdoba	37°45′24.0″N	4°49′49.8″W	283	8	1075	0.0013125
O191	Santaella, Córdoba	37°30′40.3″N	4°51′10.1″W	176	5	250	0.0017708
O192	Lopera, Jaén	37°59′08.4″N	4°15′13.0″W	195	8	106	0.0022066
O193	Andújar, Jaén	38°02′24.9″N	3°58′02.4″W	216	6	6337	0.0033242
O194	Guarromán, Jaén	38°10′54.5″N	3°42′04.7″W	351	4	53	0.0024257
O195	Ibros, Jaén	38°04′19.1″N	3°33′16.3″W	345	8	1183	0.0016047
O196	Jabalquinto, Jaén	38°01′18.8″N	3°46′27.5″W	315	9	474	0.0015316
O197	Bailén, Jaén	38°02′15.2″N	3°48′09.0″W	266	5	729	0.0014233
O198	Torre del Campo, Jaén	37°48′49.9″N	3°51′46.5″W	480	7	228	0.0027844
O199	Torredonjimeno, Jaén	37°45′16.7″N	4°07′08.3″W	381	8	1305	0.0017618
O200	El Carpio, Córdoba	37°57′07.2″N	4°30′11.1″W	137	5	1128	0.0022047
O201	Pedro Abad, Córdoba	37°58′15.6″N	4°26′19.4″W	173	6	27	0.0032630
O202	Montoro, Córdoba	38°00′27.0″N	4°17′52.4″W	167	6	97	0.0018952
O203	Montoro, Córdoba	38°01′52.1″N	4°20′19.7″W	259	6	135	0.0022104
O204	Castro del Río, Córdoba	37°41′19.3″N	4°24′12.6″W	307	5	323	0.0022815
O205	Baena, Córdoba	37°41′24.8″N	4°21′03.5″W	312	4	429	0.0024532
O206	Castro del Río, Córdoba	37°40′13.9″N	4°30′03.3″W	327	9	1635	0.0024477
O207	Moriles, Córdoba	37°25′01.4″N	4°38′29.9″W	308	5	69	0.0021067
O208	Alameda, Málaga	37°13′03.1″N	4°42′22.7″W	448	8	197	0.0022599
O209	Antequera, Málaga	37°05′37.8″N	4°33′52.4″W	435	6	324	0.0021843
O210	Antequera, Málaga	37°00′13.0″N	4°35′18.1″W	649	5	56	0.0019845
O211	Alora, Málaga	36°52′43.0″N	4°40′59.0″W	241	4	75	0.0030859
O212	Colmenar,Málaga	36°54′20.0″N	4°21′12.8″W	667	4	93	0.0036512
O213	Riogordo, Málaga	36°55′27.5″N	4°17′08.0″W	495	8	578	0.0020036
O214	La Tres Villas, Almería	37°08′55.4″N	2°43′30.9″W	763	9	702	0.0033295
**O215**	**La Tres Villas, Almería**	**37°08′15.1″N**	**2°43′28.1″W**	**706**	**7**	**1762**	0.0042730*
O216	Tabernas, Almería	37°06′07.1″N	2°16′41.7″W	533	3	121	0.0037587
**O217**	**Uleila del Campo, Almería**	**37°09′13.2″N**	**2°12′16.1″W**	**572**	**4**	**739**	0.0041255*
**O218**	**Sorbas, Almería**	**37°08′52.2″N**	**2°09′23.2″W**	**490**	**6**	**934**	0.0043722*
O219	Huércal-Overa, Almería	37°19′18.7″N	1°58′18.8″W	224	4	79	0.0024104
O220	Purchena, Almería	37°21′59.5″N	2°20′56.9″W	543	3	43	0.0033200
O221	Urrácal, Almería	37°22′30.3″N	2°21′34.3″W	592	9	507	0.0032935
O222	Armiña de Almanzora, Almería	37°21′39.2″N	2°25′33.7″W	629	3	684	0.0030571
O223	Serón, Almería	37°22′07.0″N	2°29′33.5″W	785	4	233	0.0022217
O224	Baza, Granada	37°33′00.5″N	2°44′34.5″W	700	6	501	0.0033141
O225	Baza, Granada	37°34′32.2″N	2°46′13.0″W	686	7	764	0.0030934
O226	Cortes y Graena, Granada	37°17′52.5″N	3°13′05.3″W	972	5	92	0.0015521
O227	Diezma, Granada	37°19′15.9″N	3°21′07.9″W	1282	5	64	0.0024681
O228	Alfacar, Granada	37°14′26.2″N	3°34′59.3″W	880	7	618	0.0022440
O229	Güevéjar, Granada	37°15′30.7″N	3°36′13.4″W	849	9	115	0.0022766
O230	Pinos Puente, Granada	37°11′51.2″N	3°52′19.9″W	530	5	220	0.0024649
O231	Jerez de la Frontera, Cádiz	36°44′51.4″N	6°00′24.4″W	32	7	351	0.0024601
O232	Jerez de la Frontera, Cádiz	36°46′21.3″N	5°56′52.0″W	73	4	243	0.0035819
O233	San José del Valle, Cádiz	36°34′37.9″N	5°49′15.2″W	218	5	735	0.0013023
O234	Algar, Cádiz	36°40′00.8″N	5°38′56.2″W	211	8	897	0.0025868
O235	Zahara de la Sierra, Cádiz	36°50′51.6″N	5°23′58.6″W	390	7	476	0.0028059
O236	Aldodonales, Cádiz	36°51′59.9″N	5°24′42.2″W	306	10	889	0.0024796
O237	Ecija, Sevilla	37°39′19.4″N	4°58′00.0″W	201	5	1050	0.0023624
O238	Ecija, Sevilla	37°40′31.4″N	4°59′02.9″W	183	6	143	0.0019782
O239	Marchena, Sevilla	37°16′46.4″N	5°21′51.1″W	150	5	488	0.0022139
O240	Marchena, Sevilla	37°16′05.1″N	5°21′34.1″W	148	5	188	0.0018296
O241	Marchena, Sevilla	37°15′00.8″N	5°22′09.4″W	162	7	157	0.0027083
O242	La Puebla de Cazalla, Sevilla	37°12′54.0″N	5°19′19.2″W	189	8	205	0.0032287
O243	Gibraleón, Huelva	37°21′16.7″N	7°01′15.3″W	58	7	233	0.0027071
O244	Gibraleón, Huelva	37°22′01.0″N	7°00′45.9″W	60	3	267	0.0013664
O245	Gibraleón, Huelva	37°23′22.5″N	6°55′50.0″W	67	5	312	0.0013573
O246	Beas, Huelva	37°25′06.1″N	6°47′09.8″W	109	6	318	0.0014778
O247	Beas, Huelva	37°24′09.8″N	6°45′44.8″W	82	4	132	0.0022761
O248	Beas, Huelva	37°23′33.0″N	6°44′57.8″W	67	6	177	0.0021055
**O249**	**Bollullos par del Condado, Huelva**	**37°19′22.7″N**	**6°32′47.8″W**	**101**	**6**	**1027**	0.0043603*
O250	Espiel, Córdoba	38°09′24.2″N	5°05′59.5″W	547	10	217	0.0025397
O251	San José de la Rinconada, Sevilla	37°26′18.0″N	5°50′21.4″W	41	6	156	0.0032287
O252	Huévar del Aljarafe, Sevilla	37°21′49.0″N	6°17′24.1″W	67	9	321	0.0030440
O253	Huévar del Aljarafe, Sevilla	37°21′07.1″N	6°17′45.8″W	117	5	431	0.0015883
O254	Aznalcázar, Sevilla	37°17′34.5″N	6°16′49.5″W	40	12	17481	0.0023603
O255	Bollullos de la Mitación, Sevilla	37°19′48.2″N	6°08′53.9″W	81	7	1362	0.0037966
**O256**	**Dos Hermanas, Sevilla**	**37°14′59.4″N**	**5°55′30.5″W**	**45**	**9**	**330**	0.0039641*
O257	Dos Hermanas, Sevilla	37°12′50.2″N	5°55′57.3″W	24	8	259	0.0016299
O258	El Pinar, Granada	36°54′45.8″N	3°33′56.4″W	759	4	1310	0.0017594
O259	El Valle, Granada	36°55′01.6″N	3°34′34.0″W	722	6	484	0.0022285
O260	Vegas del Genil, Granada	37°09′27.8″N	3°43′51.1″W	626	8	1269	0.0025785
O261	Las Gabias, Granada	37°08′17.5″N	3°44′05.2″W	663	4	335	0.0025266
O262	Alhama de Granada, Granada	37°08′30.4″N	3°58′40.6″W	653	7	328	0.0022782
O263	Santa Cruz del Comercio, Granada	37°04′44.6″N	3°59′31.4″W	745	7	349	0.0034424
O264	Loja, Granada	37°12′53.4″N	4°04′45.2″W	525	8	372	0.0031186
O265	Loja, Granada	37°14′12.1″N	4°04′43.9″W	543	2	33	0.0033295
O266	Utrera, Sevilla	37°13′22.6″N	5°49′08.0″W	55	12	672	0.0031654
O267	Utrera, Sevilla	37°13′48.3″N	5°49′21.7″W	59	6	541	0.0035660
O268	Utrera, Sevilla	37°06′34.5″N	5°40′27.6″W	112	5	384	0.0028069
O269	Utrera, Sevilla	37°06′36.5″N	5°40′36.1″W	86	9	257	0.0027464
O270	Utrera, Sevilla	37°07′17.3″N	5°38′08.1″W	98	6	1475	0.0029147
O271	El Arahal, Sevilla	37°11′34.3″N	5°34′15.5″W	97	6	1394	0.0022191
O272	Morón de la Frontera, Sevilla	37°07′27.0″N	5°30′15.5″W	174	6	989	0.0031236
O273	Montellano, Sevilla	37°02′28.3″N	5°33′23.4″W	196	12	1182	0.0033468
O274	Olvera, Cádiz	36°56′24.7″N	5°20′05.8″W	304	4	713	0.0033820
O275	Posadas, Córdoba	37°48′47.3″N	5°06′36.1″W	125	2	1718	0.0034729
O276	Hornachuelos, Córdoba	37°49′08.1″N	5°11′43.9″W	124	3	52	0.0035009
O277	Hornachuelos, Córdoba	37°48′00.0″N	5°14′04.6″W	85	3	62	0.0033027
O278	Peñaflor, Sevilla	37°45′01.5″N	5°19′41.1″W	170	6	156	0.0034573
O279	La Puebla de los Infantes, Sevilla	37°46′34.7″N	5°21′24.6″W	242	9	928	0.0028052
O280	La Puebla de los Infantes, Sevilla	37°47′04.5″N	5°22′21.3″W	202	10	1053	0.0018604
O281	La Puebla de los Infantes, Sevilla	37°46′39.9″N	5°23′09.2″W	262	7	1138	0.0029147
O282	La Puebla de los Infantes, Sevilla	37°46′42.7″N	5°22′01.5″W	228	4	1489	0.0016859
O283	Constantina, Sevilla	37°45′08.0″N	5°34′38.6″W	359	8	1446	0.0033929
**O284**	**Fuente Palmera, Córdoba**	**37°43′21.4″N**	**5°07′48.8″W**	**133**	**4**	**2463**	0.0044077*
O285	El Saucejo, Sevilla	37°03′11.7″N	5°04′35.2″W	580	3	110	0.0013670
O286	Alcalá del Valle, Cádiz	36°56′25.6″N	5°08′35.1″W	751	4	1112	0.0029298
O287	Alcalá del Valle, Cádiz	36°56′27.9″N	5°08′08.0″W	754	6	233	0.0027890
O288	Alcalá del Valle, Cádiz	36°53′22.8″N	5°10′58.2″W	618	7	511	0.0018271
O289	Setenil de las Bodegas, Cádiz	36°52′34.5″N	5°09′23.5″W	649	9	973	0.0024214
O290	Setenil de las Bodegas, Cádiz	36°50′48.4″N	5°13′11.5″W	776	5	183	0.0029374
O291	Ronda, Málaga	36°43′32.7″N	5°10′19.8″W	751	9	440	0.0031657
O292	Ronda, Málaga	36°47′45.7″N	5°06′23.2″W	769	5	72	0.0032048
O293	Córdoba, Córdoba	37°52′16.9″N	4°42′53.5″W	119	9	74	0.0035676
O294	Córdoba, Córdoba	37°52′45.1″N	4°42′17.2″W	103	4	224	0.0030276
O295	Adamuz, Córdoba	38°00′30.1″N	4°32′17.1″W	225	8	620	0.0025475
O296	Adamuz, Córdoba	38°03′15.6″N	4°33′01.0″W	430	7	162	0.0026257
O297	Adamuz, Córdoba	38°04′55.2″N	4°31′40.0″W	373	3	1752	0.0034213
O298	Adamuz, Córdoba	38°01′01.4″N	4°30′50.4″W	221	2	46	0.0032216
O299	Linares, Jaén	38°05′52.8″N	3°40′36.0″W	347	5	724	0.0033412
O300	Linares, Jaén	38°06′57.8″N	3°35′49.5″W	447	6	662	0.0032065
O301	Linares, Jaén	38°08′11.2″N	3°32′59.0″W	328	2	60	0.0033533
O302	Vilchez, Jaén	38°08′47.3″N	3°31′31.6″W	308	5	4232	0.0032984
O303	Arquillos, Jaén	38°11′13.0″N	3°25′26.6″W	393	4	606	0.0014655
O304	Navas de San Juan, Jaén	38°11′02.9″N	3°21′33.8″W	511	3	1136	0.0022111
O305	Úbeda, Jaén	38°07′55.0″N	3°21′33.3″W	370	5	114	0.0016853
O306	Úbeda, Jaén	38°04′04.3″N	3°13′25.8″W	723	5	134	0.0020831
O307	Sabiote, Jaén	38°05′30.0″N	3°09′46.6″W	666	4	1426	0.0014770
O308	Iznatoraf, Jaén	38°08′42.1″N	3°01′57.8″W	826	4	1572	0.0029243
O309	Beas de Segura, Jaén	38°16′11.3″N	2°57′41.1″W	532	6	1051	0.0020722
O310	Arroyo del Ojanco, Jaén	38°17′51.2″N	2°56′15.7″W	520	6	189	0.0018165
O311	Génave, Jaén	38°25′55.9″N	2°43′26.3″W	867	4	91	0.0021150
O312	Génave, Jaén	38°26′43.8″N	2°41′36.4″W	1104	4	48	0.0026945
O313	Benatae, Jaén	38°22′07.9″N	2°41′15.1″W	634	9	120	0.0016854
O314	La Iruela, Jaén	37°56′35.0″N	2°57′27.1″W	925	5	690	0.0028353
O315	Quesada, Jaén	37°50′36.2″N	3°05′28.2″W	836	6	196	0.0019142
O316	Huesa, Jaén	37°44′23.0″N	3°04′50.4″W	464	6	226	0.0018841
O317	Hinojares, Jaén	37°43′04.3″N	2°58′48.5″W	773	4	850	0.0033486
O318	Pozo Alcón, Jaén	37°44′01.5″N	2°55′19.8″W	981	3	77	0.0027161
O319	Castril, Granada	37°47′45.6″N	2°52′17.1″W	1109	7	839	0.0033614
O320	Huéscar, Granada	37°48′43.3″N	2°35′06.2″W	956	7	302	0.0031865
O321	Huéscar, Granada	37°50′20.9″N	2°31′58.4″W	988	5	1390	0.0019246
O322	Prado del Rey, Cádiz	36°46′44.6″N	5°33′31.3″W	356	6	491	0.0024043
O323	Fernán Núñez, Córdoba	37°41′44.0″N	4°44′54.5″W	251	4	881	0.0022497
O324	Lucena, Córdoba	37°24′11.4″N	4°31′47.1″W	404	11	1677	0.0024988
O325	Lucena, Córdoba	37°21′52.1″N	4°29′22.6″W	529	8	642	0.0024923
O326	Rute, Córdoba	37°23′28.1″N	4°24′49.9″W	630	7	323	0.0025106
O327	Rute, Córdoba	37°21′48.3″N	4°24′48.8″W	519	9	1546	0.0019566
O328	Iznájar, Córdoba	37°19′10.4″N	4°18′16.9″W	635	9	717	0.0017413
O329	Iznájar, Córdoba	37°16′07.1″N	4°18′26.6″W	461	10	359	0.0034249
O330	Iznájar, Córdoba	37°17′25.5″N	4°16′42.8″W	514	6	310	0.0014768
O331	Algarinejo, Granada	37°19′46.1″N	4°14′08.7″W	794	6	361	0.0027775
O332	Priego de Córdoba, Córdoba	37°25′37.0″N	4°12′19.2″W	751	7	210	0.0022001
O333	Lucena, Córdoba	37°34′09.7″N	4°13′09.8″W	451	5	19796	0.0035227
O334	Lucena, Córdoba	37°33′58.3″N	4°13′13.9″W	459	5	355	0.0029172
O335	Alcaudete, Jaén	37°35′44.6″N	4°07′57.7″W	501	6	276	0.0033196
O336	Alcaudete, Jaén	37°34′54.9″N	4°06′15.0″W	569	5	745	0.0033213
O337	Alcalá la Real, Jaén	37°27′23.9″N	3°56′36.9″W	881	8	742	0.0029092
O338	Alcalá la Real, Jaén	37°27′15.7″N	3°53′03.2″W	889	5	1551	0.0020559
O339	Alcalá la Real, Jaén	37°26′36.6″N	3°49′46.2″W	911	5	848	0.0030853
O340	Colomera, Granada	37°25′52.9″N	3°42′34.1″W	898	7	859	0.0033901
O341	Benalúa de las Villas, Granada	37°26′55.7″N	3°38′54.0″W	871	6	267	0.0021660
O342	Noalejo, Jaén	37°30′45.3″N	3°38′13.2″W	971	4	126	0.0027478
O343	Noalejo, Jaén	37°32′59.0″N	3°38′30.1″W	899	5	1168	0.0028103
O344	Cambil, Jaén	37°38′41.1″N	3°36′42.6″W	671	4	745	0.0033561
O345	Cambil, Jaén	37°38′32.3″N	3°36′06.0″W	767	3	979	0.0020844
O346	Pegalajar, Jaén	37°43′28.3″N	3°40′09.0″W	577	5	977	0.0020730
O347	Herrera, Sevilla	37°20′08.7″N	4°51′15.5″W	306	4	134	0.0016900
O348	Marinaleda, Sevilla	37°18′43.9″N	4°53′05.1″W	421	5	987	0.0023478
O349	Aguadulce, Sevilla	37°15′52.0″N	4°57′31.9″W	346	5	734	0.0023742
O350	Gilena, Sevilla	37°15′27.4″N	4°55′58.8″W	428	5	537	0.0027607
O351	Martín de la Jara, Sevilla	37°07′38.7″N	4°57′00.9″W	430	4	1988	0.0031195
O352	Sierra de Yeguas, Málaga	37°07′39.6″N	4°54′39.6″W	441	4	1197	0.0017748
O353	Campillos, Málaga	37°05′26.3″N	4°52′03.3″W	491	4	144	0.0027126
O354	Bobadilla, Málaga	37°03′11.8″N	4°45′13.6″W	412	6	505	0.0035049
O355	Bujalance, Córdoba	37°54′20.4″N	4°25′00.8″W	251	4	176	0.0018113
O356	Arjona, Jaén	37°56′04.2″N	4°04′36.4″W	403	5	151	0.0023032
O357	Montilla, Córdoba	37°34′03.8″N	4°36′32.5″W	339	9	597	0.0021325
O358	Cabra, Córdoba	37°33′17.0″N	4°30′25.5″W	550	8	406	0.0020063
O359	Cabra, Córdoba	37°30′45.8″N	4°24′40.5″W	558	9	316	0.0017762
O360	Luque, Córdoba	37°32′31.7″N	4°16′05.7″W	660	10	880	0.0017256
O361	Baena, Córdoba	37°42′29.2″N	4°21′31.1″W	381	3	164	0.0021307
O362	Córdoba, Córdoba	37°51′33.8″N	4°21′58.8″W	316	7	479	0.0024839
O363	Paterna del Campo, Huelva	37°28′32.3″N	6°25′03.4″W	122	10	283	0.0026974
O364	Paterna del Campo, Huelva	37°28′50.3″N	6°29′59.9″W	190	7	2152	0.0027154
O365	Dúrcal, Granada	37°01′01.4″ N	3°34′30.1″W	904	2	168	0.0020912
O366	Nigüelas, Granada	36°58′09.8″N	3°32′30.8″W	828	5	79	0.0022904
**O367**	**Ugíjar, Granada**	**36°58′21.0″N**	**3°00′43.4″W**	**512**	**4**	**57**	0.0040525*
O368	Padules, Almería	37°00′06.2″N	2°47′01.4″W	792	6	788	0.0012290
**O369**	**Alhabia, Almería**	**36°59′02.7″N**	**2°35′15.9″W**	**267**	**3**	**854**	0.0039660*
O370	Tabernas, Almería	37°04′56.6″N	2°17′11.4″W	522	6	854	0.0035642
O371	Úbeda, Jaén	37°59′51.3″N	3°22′57.5″W	637	4	153	0.0016660
O372	Úbeda, Jaén	37°57′45.9″N	3°19′15.9″W	389	5	576	0.0026984
O373	Jódar, Jaén	37°47′47.5″N	3°21′20.1″W	790	3	277	0.0018790
O374	Cabra del Santo Cristo, Jaén	37°39′29.5″N	3°16′40.5″W	1041	4	304	0.0023654
O375	Alicún de Ortega, Granada	37°37′21.1″N	3°08′50.3″W	710	4	128	0.0021548
O376	Morelábor, Granada	37°27′47.1″N	3°17′17.6″W	1017	7	901	0.0029735

Values in bold with * note significant contribution to beta diversity (*p* < 0.05) according to Legendre & De Cáceres [12].

a Mean altitude measured at the scale of the olive orchard in meters.

b LCBD: Local Contribution to Beta Diversity.

**Table 2:** Plant-parasitic nematode species identified in cultivated olive in Andalusia (southern Spain).

Nematode species^a^	Species	Feeding	Prevalence	Nematode abundance			Biomass^d^	SCBD^e^
	code	Habits^b^	(%)^c^	Mean	Min	Max		
*1. Aglenchus agricola*	S001	microherviborous	17.8	12.9	2	74	0.091	0.002125
*2. Amplimerlinius icarus*	S002	migratory ectoparasite	3.5	17.1	2	56	3.095	0.010993
*3. Amplimerlinius magnistylus*	S003	migratory ectoparasite	0.3	3	3	3	3.292	0.000136
*4. Amplimerlinius paraglobigerus*	S004	migratory ectoparasite	1.1	4	2	6	0.350	0.000810
*5. Aorolaimus perscitus*	S007	migratory ectoparasite	4.2	27	1	287	0.755	0.006878
*6. Aorolaimus* sp.	S008	migratory ectoparasite	0.3	8	8	8	0.755	0.000026
*7. Basiria* sp.	S010	microherviborous	1.3	13.4	2	49	0.168	0.000322
*8. Bitylenchus hispaniensis*	S011	migratory ectoparasite	13.0	51.0	3	612	0.196	0.011619
*9. Bitylenchus maximus*	S012	migratory ectoparasite	0.5	49	2	96	0.667	0.001768
*10. Coslenchus alacinatus*	S013	microherviborous	3.2	6.7	3	12	0.099	0.000312
*11. Coslenchus costatus*	S014	microherviborous	4.8	12.2	3	34	0.107	0.001139
*12. Coslenchus indicus*	S015	microherviborous	0.3	3	3	3	0.108	0.000002
*13. Criconema annuliferum*	S016	migratory ectoparasite	10.1	29.8	1	224	0.943	0.016206
*14. Criconemoides informis*	S019	migratory ectoparasite	20.5	19.4	2	181	0.608	0.017114
*15. Criconemoides morgensis*	S020	migratory ectoparasite	0.3	210	210	210	0.740	0.001339
*16. Criconemoides sphaerocephalum*	S023	migratory ectoparasite	2.7	10.6	1	28	0.317	0.000773
*17. Criconemoides xenoplax*	S024	migratory ectoparasite	5.6	103.3	2	924	0.813	0.015797
*18. Ditylenchus dipsaci*^f^	S029	migratory endoparasite	4	4.5	1	14	1.320	0.002969
*19. Ditylenchus* sp.^f^	S031	microherviborous	10.9	4	1	12	0.588	0.006853
*20. Dolichorhynchus lamelliferus*	S033	migratory ectoparasite	0.3	7	7	7	0.705	0.000006
*21. Dolichorhynchus parvus*	S035	migratory ectoparasite	1.6	126	1	506	0.091	0.003372
*22. Dolichorhynchus* sp 1	S036	migratory ectoparasite	2.1	35.4	3	112	0.499	0.004466
*23. Dolichorhynchus* sp 3	S038	migratory ectoparasite	0.3	38	38	38	0.487	0.000774
*24. Globodera* sp. *^f^	S049	sedentary endoparasite	0.3	1	1	1	0.114	0.000002
*25. Gracilacus steineri*	S051	migratory ectoparasite	1.6	28	5	71	0.033	0.000084
*26. Gracilacus straeleni*	S052	migratory ectoparasite	0.5	21	11	31	0.054	0.000081
*27. Helicotylenchus canadensis*	S053	migratory ectoparasite	1.6	337.3	14	1964	0.586	0.011342
*28. Helicotylenchus digonicus*	S054	migratory ectoparasite	48.1	485.3	2	7120	0.247	0.171107
*29. Helicotylenchus exallus*	S055	migratory ectoparasite	2.4	37.3	12	119	0.244	0.002366
*30. Helicotylenchus microlobus*	S056	migratory ectoparasite	2.4	710	14	3076	0.336	0.018585
*31. Helicotylenchus minzi*	S057	migratory ectoparasite	0.5	23	13	33	0.272	0.002041
*32. Helicotylenchus oleae*	S058	migratory ectoparasite	20.2	599.9	7	19720	0.145	0.079150
*33. Helicotylenchus* sp 1	S060	migratory ectoparasite	0.3	2312	2312	2312	0.347	0.003498
*34. Helicotylenchus* sp 4	S063	migratory ectoparasite	0.3	4	4	4	0.333	0.000327
*35. Helicotylenchus* sp 5	S064	migratory ectoparasite	0.3	14	14	14	0.326	0.000430
*36. Helicotylenchus vulgaris*	S065	migratory ectoparasite	18.9	268	4	1092	0.662	0.116413
*37. Hemicriconemoides macrodorus*	S066	migratory ectoparasite	2.1	45.7	1	112	0.714	0.006267
*38. Hemicycliophora iberica*	S070	migratory ectoparasite	0.3	2	2	2	0.572	0.000099
39. Heterodera avenae*^f^	S077	sedentary endoparasite	2.4	43.5	2	345	0.154	0.000161
40. Heterodera mediterranea*	S081	sedentary endoparasite	1.6	22.3	5	46	0.104	0.002558
*41. Heterodera* sp. *^f^	S083	sedentary endoparasite	0.3	4	4	4	0.102	0.000026
*42. Longidorus alvegus*	S087	migratory ectoparasite	0.5	6.5	1	12	6.302	0.002652
*43. Longidorus indalus*	S091	migratory ectoparasite	1.6	11.2	1	52	3.794	0.005117
*44. Longidorus macrodorus*	S261	migratory ectoparasite	0.3	1	1	1	72.799	0.003572
*45. Longidorus magnus*	S095	migratory ectoparasite	0.8	1.7	1	2	72.453	0.003382
*46. Longidorus oleae*	S096	migratory ectoparasite	0.5	2.5	2	3	37.027	0.000489
*47. Longidorus rubi*	S098	migratory ectoparasite	0.3	2	2	2	52.429	0.000256
*48. Longidorus vineacola*	S104	migratory ectoparasite	0.3	1	1	1	17.890	0.003057
*49. Longidorus vinearum*	S105	migratory ectoparasite	0.3	3	3	3	58.502	0.001073
*50. Longidorus wicuolea*	S106	migratory ectoparasite	0.3	5	5	5	28.861	0.000842
51. Meloidogyne arenaria*	S107	sedentary endoparasite	0.8	59.7	2	138	0.068	0.000024
52. Meloidogyne artiellia*^f^	S108	sedentary endoparasite	0.5	11.5	9	14	0.054	0.000002
53. Meloidogyne hapla*	S110	sedentary endoparasite	0.3	2	2	2	0.088	0.000002
54. Meloidogyne incognita*	S112	sedentary endoparasite	0.8	1951.7	4	5727	0.049	0.003219
55. Meloidogyne javanica*	S113	sedentary endoparasite	3.7	781.4	1	10000	0.068	0.006666
*56. Meloidogyne* sp 1 *	S114	sedentary endoparasite	0.5	25	4	46	0.071	0.000125
*57. Merlinius brevidens*	S116	migratory ectoparasite	72.6	35.8	2	176	0.185	0.039512
*58. Nagelus obscurus*	S118	migratory ectoparasite	0.3	28	28	28	0.137	0.000178
*59. Neodolichorhynchus microphasmis*	S122	migratory ectoparasite	0.3	42	42	42	0.460	0.001402
*60. Neopsilenchus* sp.	S124	microherviborous	0.3	31	31	31	0.188	0.000211
*61. Ogma civellae*	S126	migratory ectoparasite	1.1	13.2	3	38	1.033	0.003519
*62. Ogma palmatum*	S128	migratory ectoparasite	0.3	6	6	6	1.175	0.000424
*63. Ogma rhombosquamatum*	S129	migratory ectoparasite	20.5	483.3	1	9800	0.569	0.018593
*64. Paratrichodorus “allius”*	S131	migratory ectoparasite	2.7	5.2	1	12	1.324	0.001412
*65. Paratrichodorus* sp 1	S134	migratory ectoparasite	0.8	9.7	3	14	0.291	0.000656
*66. Paratrichodorus* sp 10	S135	migratory ectoparasite	0.3	4	4	4	0.645	0.000039
*67. Paratrichodorus* sp 15	S138	migratory ectoparasite	0.3	18	18	18	0.685	0.000096
*68. Paratrichodorus* sp 3	S140	migratory ectoparasite	0.3	3	3	3	0.633	0.000149
*69. Paratrichodorus* sp 4	S141	migratory ectoparasite	0.8	1.7	1	3	0.622	0.000096
*70. Paratrichodorus* sp 6	S143	migratory ectoparasite	0.3	15	15	15	0.698	0.000249
*71. Paratrichodorus* sp 9	S145	migratory ectoparasite	0.3	3	3	3	0.614	0.000010
*72. Paratrophurus loofi*	S147	migratory ectoparasite	1.9	14.9	3	56	0.748	0.004933
*73. Paratrophurus striatus*	S148	migratory ectoparasite	0.3	12	12	12	0.295	0.000156
*74. Paratylenchus ciccaronei*	S150	migratory ectoparasite	6.6	132.2	3	974	0.047	0.002823
*75. Paratylenchus microdorus*	S151	migratory ectoparasite	23.1	185.1	2	7480	0.074	0.014765
*76. Paratylenchus sheri*	S152	migratory ectoparasite	8.5	347.4	4	3024	0.109	0.019067
*77. Paratylenchus vandenbrandei*	S153	migratory ectoparasite	13.6	137.7	4	1736	0.027	0.013655
*78. Pratylenchoides alkani*	S154	migratory endoparasite	0.8	29	2	49	0.594	0.000389
*79. Pratylenchoides crenicauda*	S155	migratory endoparasite	0.3	38	38	38	0.310	0.000119
*80. Pratylenchoides hispaniensis*	S156	migratory endoparasite	0.3	2	2	2	0.482	0.000006
*81. Pratylenchoides ritteri*	S157	migratory endoparasite	0.5	68.5	48	89	0.408	0.001098
*82. Pratylenchus neglectus*^f^	S159	migratory endoparasite	6.4	10.8	2	47	0.097	0.000717
*83. Pratylenchus oleae*	S160	migratory endoparasite	0.8	55	28	98	0.099	0.002373
*84. Pratylenchus penetrans*	S161	migratory endoparasite	0.8	14.3	7	19	0.171	0.000496
*85. Pratylenchus scribneri*	S162	migratory endoparasite	0.3	24	24	24	0.139	0.000186
*86. Pratylenchus thornei*^f^	S169	migratory endoparasite	17	16.8	1	126	0.148	0.007639
*87. Psilenchus hilarulus*	S172	microherviborous	7.4	11.1	1	121	0.391	0.005227
*88. Psilenchus hilarus*	S173	microherviborous	0.8	6	2	7	0.388	0.000259
89. Rotylenchulus macrosoma*	S176	semiendoparasite	0.5	508.5	141	876	0.048	0.000528
*90. Rotylenchus cypriensis*	S177	migratory ectoparasite	0.3	57	57	57	0.185	0.000407
*91. Rotylenchus incultus*	S180	migratory ectoparasite	3.5	277.5	4	1230	0.317	0.012569
*92. Rotylenchus* sp 1	S184	migratory ectoparasite	0.5	31	3	59	0.918	0.000875
*93. Rotylenchus* sp 5	S188	migratory ectoparasite	0.3	8	8	8	0.942	0.000029
*94. Trichodorus andalusicus*	S191	migratory ectoparasite	8.5	16.3	1	57	0.190	0.002358
*95. Trichodorus giennensis*	S192	migratory ectoparasite	7.7	10.9	1	58	0.235	0.002674
*96. Trichodorus onubensis*	S195	migratory ectoparasite	0.3	6	6	6	0.518	0.000339
*97. Trichodorus paragiennensis*	S196	migratory ectoparasite	0.5	11	3	19	0.497	0.000096
*98. Trichodorus parasilvestris*	S197	migratory ectoparasite	0.3	34	34	34	0.367	0.000134
*99. Trichodorus* sp. AMS-2014	S199	migratory ectoparasite	0.3	12	12	12	1.226	0.000050
*100. Trophurus imperialis*	S201	migratory ectoparasite	0.8	11	1	23	0.558	0.000891
*101. Tylenchorhynchus clarus*	S204	migratory ectoparasite	11.1	121.7	4	2072	0.108	0.014698
*102. Tylenchorhynchus laeviterminalis*	S207	migratory ectoparasite	0.8	89	10	178	0.159	0.000703
*103. Tylenchorhynchus mediterraneus*	S209	migratory ectoparasite	7.2	91.6	3	672	0.511	0.025731
*104. Tylenchorhynchus zeae*	S205	migratory ectoparasite	0.5	19.5	12	27	0.153	0.002521
*105. Tylenchus davainei*	S215	microherviborous	21.5	27	2	252	0.594	0.031589
*106. Tylenchus elegans*	S216	microherviborous	30.8	19.8	2	127	0.447	0.025444
*107. Tylenchus magnus*	S218	microherviborous	0.5	10.5	4	17	0.481	0.000132
*108. Tylenchus* sp.	S220	microherviborous	0.3	3	3	3	0.517	0.000231
*109. Xiphinema adenohystherum*	S222	migratory ectoparasite	0.8	1.7	1	2	11.346	0.001251
*110. Xiphinema baetica*	S225	migratory ectoparasite	0.3	1	1	1	9.897	0.000106
*111. Xiphinema cadavalense*	S226	migratory ectoparasite	0.5	1	1	1	20.547	0.000989
*112. Xiphinema coxi europaeum*	S230	migratory ectoparasite	1.3	6.2	1	18	7.494	0.004608
*113. Xiphinema duriense*	S231	migratory ectoparasite	0.3	1	1	1	0.666	0.000129
*114. Xiphinema hispidum*	S234	migratory ectoparasite	0.3	30	30	30	4.040	0.001856
*115. Xiphinema incertum*	S235	migratory ectoparasite	0.3	38	38	38	1.319	0.002035
*116. Xiphinema index*	S236	migratory ectoparasite	0.3	3	3	3	5.426	0.000946
*117. Xiphinema italiae*	S237	migratory ectoparasite	8.5	11.6	1	59	1.882	0.014404
*118. Xiphinema iznajarense*	S262	migratory ectoparasite	0.3	34	34	34	11.564	0.003091
*119. Xiphinema macrodora*	S239	migratory ectoparasite	0.5	11	8	14	51.391	0.004781
*120. Xiphinema mengibarense*	S263	migratory ectoparasite	0.3	21	21	21	6.359	0.001333
*121. Xiphinema nuragicum*	S241	migratory ectoparasite	9.3	21.9	1	218	9.295	0.040846
*122. Xiphinema pachtaicum*	S244	migratory ectoparasite	70.4	35.7	1	819	1.047	0.091476
*123. Xiphinema* sp 4	S253	migratory ectoparasite	0.3	1	1	1	4.308	0.001123
*124. Xiphinema* sp 5	S171	migratory ectoparasite	0.5	20	12	28	0.841	0.004496
*125. Xiphinema turcicum*	S255	migratory ectoparasite	1.3	9.6	2	22	8.885	0.007783
*126. Xiphinema turdetanense*	S256	migratory ectoparasite	0.5	2.5	1	4	8.792	0.000213
*127. Xiphinema vallense*	S257	migratory ectoparasite	0.5	14	12	16	1.084	0.000623
*128. Zygotylenchus guevarai*^f^	S258	migratory endoparasite	6.9	26.6	2	264	0.100	0.002409

a For species identification see: [6, 7, 13, 14, 15, 16].

b Feeding habits according to Yeates et al. [17].

c Prevalence was calculated as the percentage of samples in which a nematode species was diagnosed with respect to total number of samples.

d Relative nematode wet biomass according to an adjusted Andrassy's formula [10]; relative biomass (μg) = L x D2/1.600.000; where L is nematode body length (in μm). and D is nematode maximum body width (in μm). (*) Biomass based on second-stage juveniles.

e SCBD: species contribution to beta diversity [12].

f Plant-parasitic nematodes species could be associated with cultivated and wild legumes growing as cover crops rather than with cultivated olives; as olive is not a suitable host for this PPN species [4].

The diversity, prevalence and abundance of PPN associated with cultivated olive are presented in Table 2, Fig. 1, Fig. 2. Data were characterized by performing species diversity under integrative taxonomy identification at species level of PPN infesting soils from 376 sampled commercial olive orchards in Andalusia, southern Spain [1] (Table 1). Thus, 128 PPN species belonging to 38 genera and to 13 families were recorded, which highlights a high taxonomical diversity of PPN communities. However, it should be pointed out that species belonging to genus *Filenchus* were not included because of its feeding habits as plant feeding are not fully clarified [2]. Other PPN species such as *Heterodera avenae*, *Pratylenchus neglectus*, *Pratylenchus thornei*, *Zygotylenchus guevarai* or other species from the genera *Ditylenchus*, *Heterodera* and *Globodera* were included in the analysis although olive is not a suitable host for them but they were detected from the rhizosphere of olive tree and could be associated with host plants growing as cover crops in the orchards. The nematode abundance in each commercial olive orchard ranged from 7 to (O31) to 19,796 (O333) nematode specimens per 500 cm^3^ of soil [1] (Table 1). The number of PPN species per nematode family ranged from one in the case of the family Rotylenchulidae to 28 species for the family Longidoridae. Other families comprising species among the most damaging plant pathogens worldwide such as Meloidogynidae encompassed six sedentary endoparasite nematodes species (*Meloidogyne* spp.). The three most prevalent families were Tylenchidae, Paratylenchidae and Criconematidae, and the nematodes families with the highest average nematode densities were Meloidogynidae, Hoplolaimidae and Paratylenchidae. In fact, migratory ectoparasite PPN such as *Helicotylenchus oleae* and *Ogma rhombosquamatum* showed the highest nematode abundance (19,720 and 9800 nematodes per 500 cm^3^ of soil, respectively); however, a rare (low prevalence) of sedentary endoparasitic PPN species such as *Meloidogyne javanica* was also detected at a high nematode abundance, i.e. 10,000 nematodes per 500 cm^3^ of soil. The species prevalence ranged from 0.3 (several nematodes species detected only in one sampling site) to 72.6% (*Merlinius brevidens*). Data revealed a remarkable diversity of PPN associated with olive trees, which agrees with the fact described that olive acts as host plant of a large variety of PPN [3,4]. Data increase the number of PPN associated with olive trees, being estimated in about 250 species documented worldwide [[3], [4], [5], [6], [7]]. The common genera of PPN observed were similar to those reported in previous surveys in olive trees in Andalusia [5] and Morocco [3] except for the remarkable taxonomical diversity detected for the family Longidoridae (28 species). Data also showed the nematode biomass for species of PPN identified. SCBD values ranged from almost zero to 17% for the migratory ectoparasitic PPN species *Helicotylenchus digonicus* (Table 2).

## 2 Experimental design, materials, and methods

### 2.1 Sampling design

Data was obtained by systematic survey based on sampling design described by Archidona-Yuste et al. [1]. A total of 376 commercial olive orchards were selected across the entire olive area of Andalusia (Table 1). In brief, soil samples were collected from 2011 to 2016 during the spring season. In each commercial olive orchard, soil samples were taken from four to five healthy-looking trees that were georeferenced. Soil samples were collected with a hoe discarding the upper 5-cm top soil profile, from a 5- to 50-cm depth, in the close vicinity of active olive roots. In fact, we ensured that roots from other plants including weeds or other herbaceous plants were not included. Finally, all individual samples were thoroughly mixed to obtain a single representative sample per each commercial olive orchard before nematode extraction and physicochemical parameters determination [1].

### 2.2 Nematode extraction

From each soil sample, nematodes were extracted separately from two 250-cm^3^ subsamples using magnesium sulfate centrifugal-flotation method [6,8]. Soil was washed thoroughly with tap water through a 710-μm mesh sieve, and the filtered water was collected in a beaker and extensively mixed with 4% kaolin (v/v). This mixture was centrifuged at 1100×*g* for 4 min, and the supernatants discarded. Pellets were re-suspended in 250 ml MgSO4 (δ = 1.16) and the new suspensions were centrifuged at 1100×*g* for 3 min. The supernatants were sieved through a 5 μm mesh, and nematodes collected on the sieve were washed with tap water [4]. Water solution containing nematodes collected from each of the two 250 cm^3^ were mixed in a single one in order to carry out the diagnostic and identification of nematodes from a 500 cm^3^ soil subsample.

### 2.3 Nematode identification

In order to select the PPN from the global nematode community in the soil, the nematode sample was poured into a counting dish (8 cm L x 8 cm W x 1.5 cm H), where they were identified and then, counted under a stereo-microscope (Leica MZ12; Leica Microsystems, Wetzler, Germany). PPN were identified to genus, and then we focused on the species delineation selecting adult nematode specimens which were fixed in a solution of 4% formaldehyde +1% propionic acid and processed to pure glycerine using Seinhorst's method [9], and identified by morphological traits and molecular markers to species level. The morphological study at nematode species level was performed by classical diagnostic features using general and specific taxonomic keys from each nematode family and genus. However, the identification of nematode species based solely on morphological diagnostic is quite complex due to the occurrence of cryptic species and/or overlapping of morphological diagnostic characters among PPN species [[5], [6], [7]]. Therefore, polyphasic identification, based on an integrative taxonomy of combining both molecular and morphological techniques, was performed to get an efficient and reliable identification of PPN species (*see Notes* in Table 2).

### 2.4 Prevalence, abundance, biomass and species richness calculation

Prevalence was calculated by dividing the number of samples in which PPN species was detected by the total number of samples and expressed as a percentage. Total nematode abundance in each commercial orchard was calculated as the total number of specimens from all species identified per 500 cm^3^ of soil for each commercial olive orchard. For each species identified, the abundance was calculated as the total number of specimens per 500 cm^3^ of soil. Relative nematode individual fresh biomass was calculated according to an adjusted Andrassy's formula [10], wherein relative biomass (μg) = L x D^2^ * 1,600,000^−1^; where L is nematode body length (in μm), and D is nematode maximum body width (in μm). Nematode size was determined with indications described by Archidona-Yuste et al., [1]. In addition, nematode species richness was determined for each olive orchard.
